# Supplementary material for: Identification of human skin microbiome odorants that manipulate mosquito landing behavior
Source: Sci Rep. 2024 Jan 18;14:1631. doi: 10.1038/s41598-023-50182-5 (PMC10796395; doi:10.1038/s41598-023-50182-5)
Supplement: Supplementary file 1 — Supplementary Figure S1. [file 41598_2023_50182_MOESM1_ESM.pdf]

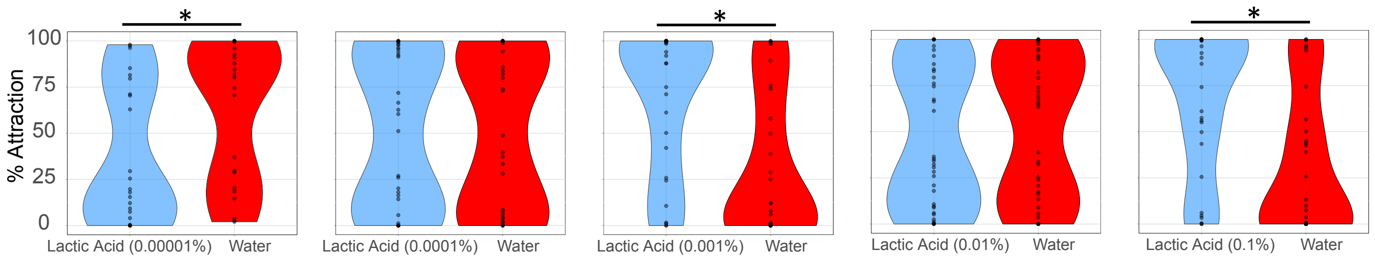

Supplemental Figure S1. Two-choice landing assays with skin odorants. Dose-response assays were performed, as shown in Figure 1, between three and five different concentrations, using the L-(+)-lactic acid as testing odorant as the tested odorant. Statistically significant differences at $p < 0.05$ are indicated by an asterisk (*). n = 3 biological replicates, for which the behavior activity of individual mosquitoes was recorded and represented by each dot. Plots represent pooled data of the biological replicates.
